# Relationship between the number of lost teeth and the occurrence of depressive symptoms in middle-aged adults: a cross-sectional study

**DOI:** 10.1186/s12903-024-04337-z

**Published:** 2024-05-13

**Authors:** Martyna Głuszek–Osuch, Elżbieta Cieśla, Edyta Suliga

**Affiliations:** Institute of Health Sciences, Collegium Medicum, Jan Kochanowski University, Kielce, Poland

**Keywords:** Depressive symptoms, Tooth loss, Middle-aged adults

## Abstract

**Background:**

Many recent studies suggest the existence of a relationship between oral health and the occurrence of depressive symptoms. The aim of this study was to assess the relationship between the number of lost teeth and the occurrence of depressive symptoms in middle-aged adults.

**Methods:**

An analysis was performed on the data obtained from the PONS project (POlish-Norwegian Study), conducted in the Świętokrzyskie Province in Poland in 2010–2011. The research material included the cross-sectional data of 11,901 individuals aged 40–64 years (7967 women). Depressive symptoms, used as outcome variables, were assessed with a questionnaire. The participants provided the responses to questions concerning the occurrence of eight symptoms over the last 12 months. The answers were scored as 1 point or 0 points. The participants were divided into three tercile groups based on their total scores: no or mild (0–2 points), moderate (3–5 points), and severe depressive symptoms (6–8 points). The self-reported number of lost teeth was analysed according to the following categories: 0–4, 5–8, 9–27, and a complete lack of natural teeth. Multivariable logistic regression analysis for depressive symptoms was used in relation to the number of lost teeth. The following covariates were included in the adjusted model: age, sex, place of residence, education, marital status, BMI, diabetes status, stressful life events in the last year, use of antidepressants, smoking, and sugar and sweet consumption.

**Results:**

The likelihood of both moderate (OR = 1.189; 95%CI: 1.028–1.376; *p* < .020) and severe (OR = 1.846; 95%CI: 1.488–2.290; *p* < .001) depressive symptoms showed the strongest relationship with a total lack of natural teeth. A loss of more than 8 natural teeth was also significantly associated (OR = 1.315; 95%CI: 1.075–1.609; *p* < .008) with the occurrence of severe depressive symptoms.

**Conclusions:**

The loss of natural teeth was positively related to the occurrence of depressive symptoms in middle-aged adults. Thus, there is an urgent need to intensify stomatological prophylaxis, education and treatment for middle-aged individuals.

## Introduction

Depression is one of the most common mental health disorders worldwide and can originate from genetic, biological, and environmental factors [1, 2]. Depressive symptoms, such as lowered mood (sadness, despondency, reliving of negative events, anhedonia, and indifference); decreased psychomotor drive (psychomotor retardation or inhibition, loss of energy, and persistent fatigue); abnormal circadian rhythm; and somatic symptoms (hyposomnia, hypersomnia, dry mucous membranes in the mouth, and weight changes), may also occur in people who do not meet the clinical diagnostic criteria for depression and who experience subthreshold depression. Many recent studies suggest the existence of a relationship between oral health and the occurrence of depressive symptoms [3, 4]. One of the most severe oral conditions is tooth loss [5, 6]. Its direct causes are usually untreated caries and periodontal diseases [7–9]. The prevalence of tooth loss and the factors that directly determine it depend largely on economic development, access to dental care, social factors, and lifestyle, which is why they may differ between countries and regions [8–15]. Loss of teeth not only reduces the effectiveness of mastication but also contributes to a limitation, or even complete elimination, of hard products from the diet (e.g., nuts and some raw fruits and vegetables), significantly reducing the nutritional value of food [16]. Consequently, such changes may impact the nutritional status and health of individuals. Loss of teeth results in a gradual decrease in bone tissue and may lead to malocclusion and disorders of the temporomandibular joints. It also causes speech impediments, especially lisping, since without proper support in the form of teeth, the tongue cannot be positioned to correctly articulate some sounds [17]. Furthermore, visibly missing teeth constitute an aesthetic blemish, while poorly fitted dentures and incorrect articulation reduce self-confidence, make it difficult for individuals to be active in the job market, and cause individuals to withdraw from social life, which in turn may reduce mental well-being [17, 18]. These relationships have, to date, been analysed predominantly among elderly individuals, with very few publications addressing the topic among middle-aged adults. The aim of this study was to assess the relationship between the number of lost teeth and the occurrence of depressive symptoms among individuals aged 40–64 years. According to our hypothesis, the number of lost natural teeth shows a positive relationship with the occurrence of depressive symptoms among middle-aged adults.

## Materials and methods

### Study design

An analysis was performed on the cross-sectional data obtained as part of the PONS project (POlish-Norwegian Study), which was conducted in the Świętokrzyskie Province in Poland in 2010–2011. The goal of the project was to observe the health and prevalence of chronic noncommunicable diseases among the residents of southeastern Poland [19]. This was a facility-based survey. All participants of the project were volunteers. The study in its entirety (both the measurements and the interviews) was conducted in the medical institutions that took part on the project. The Ethics Committee at the Cancer Center and Institute of Oncology in Warsaw (No. 69/2009/1/2011) approved the project and method of data collection. Further data analysis was also approved by the Committee on Bioethics at the Faculty of Health Sciences, Jan Kochanowski University in Kielce (No. 45/2016).

### Participants

Participants were recruited by invitations to participate in the project to all men and women aged 45–64 years living in the Kielce District (Świętokrzyskie Province, Poland). The participants were invited to take part in the study through advertisements and promotional articles in local newspapers, radio and TV programmes, informative materials (leaflets, billboard advertising, information charts and a website), co-operation with family doctors and medical institutions participating in the project, which sent the invitations via mail. Therefore, all participants of the project were volunteers. The sole inclusion criterion for the whole project was age 45–64 years and being a resident of the city of Kielce and the surrounding rural area (the Kielce County). We did not directly calculate the sample size. We assumed a response rate of at least 10% among the target group and the actual response rate was 12%. Many more women than men from the target age group volunteered to participate. A small number of participants who were younger (37–44 years) and older (65–66 years) than the target age group also volunteered for the study. Consequently, the collected data encompassed the results of 13,172 individuals aged 37–66 years. For the purposes of the analyses conducted in this study, individuals aged ≥ 65 years and < 40 years, as well as participants whose data were incomplete, were excluded (1271 individuals). Ultimately, the research material for the study included the data of 11,901 individuals aged 40–64 years (3934 men).

### Measurements

Sociodemographic data and data related to depressive symptoms, the declared number of lost teeth, and lifestyle factors were collected via face-to-face interviews with structured questionnaires. The sociodemographic variables included sex (male or female), age (years), place of residence (the city of Kielce (200 thousand inhabitants) and the rural county of Kielecki (village), education level (higher, secondary, primary, or vocational), and marital status (married or in a stable relationship, single, or widowed/widowered). Measurements of depressive symptoms, used as outcome variables, were adapted from the Prospective Urban and Rural Epidemiological (PURE) study [20]. The participants were assessed based on the responses provided to eight questions concerning the occurrence of the following symptoms in the last 12 months: fatigue (loss of energy), weight gain or loss, problems falling asleep, loss of concentration, loss of interest and pleasure, feeling of helplessness (low self-esteem), sadness (worry), and thoughts about death. The respondents answered “yes” or “no” to each question. The answers were scored as 1 point or 0 points. The participants were then divided into three tercile groups based on their total scores (from 0 to 8 points): no or mild depressive symptoms (0–2 points), moderate depressive symptoms (3–5 points), and severe depressive symptoms (6–8 points) [21]. The respondents were also asked about the use of antidepressants and whether they had experienced any stressful life events in the last year, such as a serious disease or injury or death or serious disease of a close family member. The categories of the variable “number of lost teeth” were distinguished based on the quartile distribution of this characteristic among the analysed population. Thus, the self-reported number of lost teeth was analysed according to the following categories: 0–4, 5–8, 9–27, and a complete lack of natural teeth. Body height and mass were measured and subsequently used to calculate BMI (kg/m^2^). The assessment of diabetes status included a diagnosis of diabetes, diabetes treatment, or current abnormal fasting glucose in the blood serum (≥ 100 mg/dL/5.5 mmol/L) measured using the enzyme method with hexokinase. Participants who did not meet the above criteria were classified as nondiabetic. Total consumption of sugar, sweets, and sweetened beverages was assessed with a validated, semiquantitative food frequency questionnaire from the PURE study, which was used in the PONS project [22]. The questionnaire was administrated in Polish. The method of FFQ development and validation was carefully standardised for all PURE study participating countries [22]. One hundred and forty-six study participants in the Polish arm of the PURE study completed the 134-item FFQs as well as four 24-h dietary recalls during a 12-month period. The FFQ has good validity and reproducibility in relations to the reference method. The study participants were asked about how frequently they consumed certain portions of each product containing sugar over the last year. The frequencies of consumption were classified as follows: 6 times a day or more, 4–5 times a day, 2–3 times a day, once a day, 5–6 times a week, 2–4 times a week, once a week, 1–3 times a month, less frequently than once a month or not at all. The reported frequencies of consumption and sizes of portions of the analysed food products were converted into mean daily doses (min–max = 0.00–27.64 times/day). Smoking status was determined by dividing the respondents into current smokers (smoking every day), former smokers (those who had not smoked for more than six months at the time of the study), and never smokers (the remaining respondents).

### Statistical analysis

**Fig. 1:**
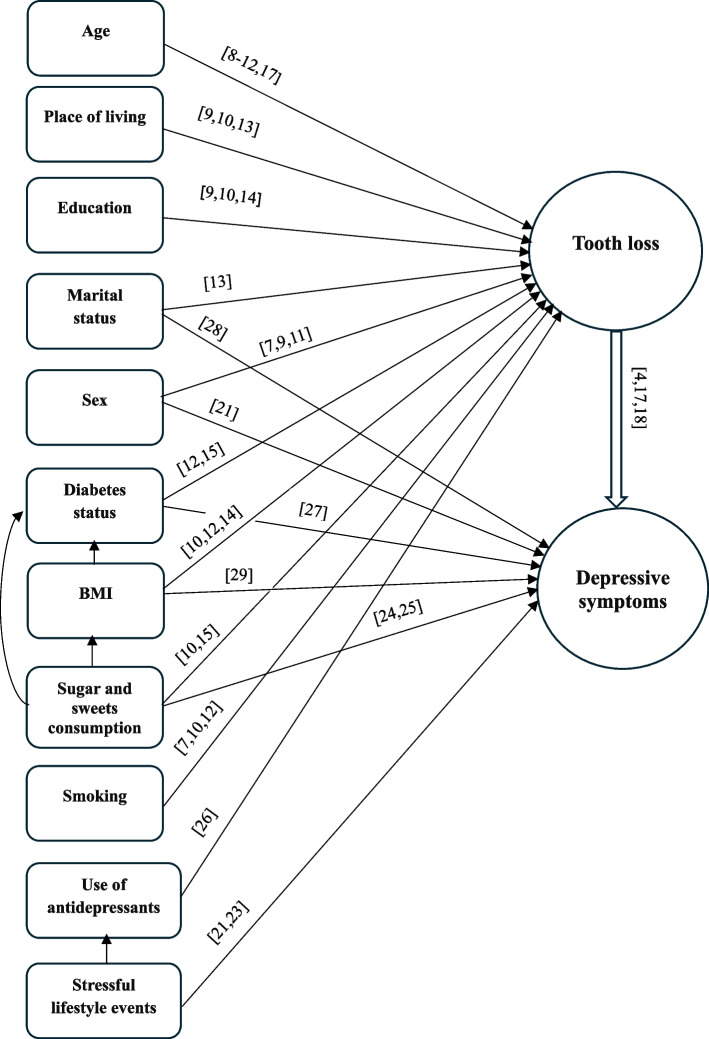
Relationship between dependent and independent variables and confounders [7–15, 17, 18, 21, 23–29]

The data were analysed using STATISTICA 13.3 software (STATSOFT, PL). The results were considered significant at *p* ≤ 0.05. Counts and percentages were calculated for each categorical (qualitative) variable. The arithmetic mean (*X*) and standard deviation (*SD*) were calculated for the continuous variables, i.e., age, BMI, and sugar and sweets consumption. The internal consistency and reliability of the eight-question test used to assess depressive symptoms were calculated based on Cronbach’s alpha, which equaled 0.81 in this sample. Simple and multivariable logistic regression analyses were conducted to calculate odds *ratios (ORs*) and 95% confidence *intervals (CIs*). The number of lost teeth was an independent variable (ref. 0–4). Depressive symptoms were analysed according to three categories, with moderate (3–5 points) and severe (6–8 points) symptoms forming the model categories and no or mild (0–2 points) symptoms forming the reference group (ref.). Multivariable models were calculated adjusted for the following covariates: age and consumption of sugar and sweets (continuous variables) and categorical variables: sex (ref. women), place of residence (ref. city), education (ref. higher), marital status (ref. in a relationship), BMI (ref. 18.5–24.9 kg/m^2^), diabetes status (ref. no), stressful life events in the last year (ref. no), use of antidepressants (ref. no), and smoking (ref. never smokers). The goodness-of-fit model was assessed using the Akaike Information Criterion (AIC). The covariates were selected based on a review of the subject literature [7–15, 17, 18, 23–29] and a previous study [21] (Fig. 1).

## Results

Table 1 presents the sociodemographic and clinical data of the participants. The majority of the participants were women (63.89%), lived in a city, had secondary education, and were in a stable relationship. The mean BMI was 28.14 ± 4.65 kg/m^2^, and diabetes or elevated glucose was observed in more than 1/3 of the participants. In terms of the number of lost teeth, the largest group included individuals who lost 9 or more teeth (28.56%). The mean intensity of depressive symptoms was 2.22 ± 2.29 points. Only 2.91% of the participants used antidepressants regularly. Over 22% declared experiencing stressful life events in the last year. The mean total consumption of sugar, sweets, and sweetened beverages was 4.32 ± 3.60 times/day. Almost 1/5 of the participants were current smokers.

**Table 1:** Sociodemographic and clinical characteristics of the respondents (*N* = 11,901)

Variables			*N* (%)
Sociodemographic	Sex	Men	3934 (33.1)
		Women	7967 (63.9)
	Age (years)	*X* ± *SD*	55.64 ± 5.3
	Place of residence	City	7414 (62.3)
		Village	4487 (37.7)
	Education	Higher	3127 (26.3)
		Secondary	5310 (44.6)
		Primary or vocational	3464 (29.1)
	Marital status	In a relationship	9441 (79.3)
		Single	2460 (20.7)
Clinical and health	BMI (kg/m^2^)	*X* ± *SD*	28.14 ± 4.65
		< 18.5	51 (0.5)
		18.5–24.9	3068 (28.2)
		25.0–29.9	5198 (43.2)
		≥ 30.0	3584 (28.2)
	Diabetes status	No	7941 (66.7)
		Yes	3960 (33.3)
	Number of lost teeth	0–4	2908 (24.5)
		5–8	2932 (24.6)
		9–27	3399 (28.6)
		No natural teeth	2662 (22.4)
	Depressive symptoms	*X* ± *SD* (points)	2.22 ± 2.29
		None or mild (0–2 points)	7469 (62.8)
		Moderate (3–5 points)	3038 (25.5)
		Severe (6–8 points)	1394 (11.7)
	Use of antidepressants	No	11555 (97.1)
		Yes	346 (2.9)
	Stressful life events in the last year	No	9255 (77.8)
		Yes	2646 (22.2)
Lifestyle	Sugar and sweets consumption (times/day)	*X* ± *SD*	4.32 ± 3.60
	Smoking	Never	5588 (47.0)
		Former	3996 (33.6)
		Current	2317 (19.5)

According to the unadjusted model, a greater number of lost teeth was associated with a greater likelihood of both moderate and severe depressive symptoms (Table 2). After adjusting for the confounding variables, the relationships between the number of lost teeth and depressive symptoms were weaker, but a total lack of natural teeth was still the factor that was most strongly connected with the likelihood of both moderate (*p* < 0.020) and severe (*p* < 0.001) symptoms (Table 3). A loss of more than 8 natural teeth was also significantly associated (*p* < 0.008) with the occurrence of severe depressive symptoms.

**Table 2:** Simple multinomial logistic regression analysis of moderate and severe depressive symptoms in relation to the number of lost teeth

Depressive symptoms	Number of lost teeth	OR (95% CI)	p
Moderate vs. none or mild	0–4	Ref	
	5–8	1.129 (0.999—1.276)	0.051
	9–27	1.346 (1.197—1.513)	** < 0.001**
	No natural teeth	1.474 (1.301—1.669)	** < 0.001**
Severe vs. none or mild	0–4	Ref	
	5–8	1.342 (1.116—1.613)	**0.002**
	9–27	1.854 (1.562—2.202)	** < 0.001**
	No natural teeth	2.624 (2.207—3.120)	** < 0.001**

Akaike Information Criterion – AIC = 16,201.74

*OR (95% CI)* Odds Ratio and 95% Confidence Interval

**Table 3:** Multivariable multinomial logistic regression analysis of depressive symptoms in relation to the number of lost teeth^a^

Depressive symptoms	Number of lost teeth	OR (95% CI)	p
Moderate vs. none or mild	0–4	Ref	
	5–8	1.052 (0.926—1.196)	0.437
	9–27	1.132 (0.993—1.291)	0.064
	No natural teeth	1.189 (1.028—1.376)	**0.020**
Severe vs. none or mild	0–4	Ref	
	5–8	1.165 (0.951—1.426)	0.141
	9–27	1.315 (1.075—1.609)	**0.008**
	No natural teeth	1.846 (1.488—2.290)	** < 0.001**

Akaike Information Criterion – AIC = 15076.62

*OR (95% CI)* Odds Ratio and 95% Confidence Interval

^a^adjusted for sex, age, place of residence, education, marital status, BMI, diabetes status, use of antidepressants, stressful life events in the last year, sugar and sweets consumption, and smoking

## Discussion

The obtained results confirmed the hypothesis about the existence of a positive relationship between the number of lost teeth and the occurrence of depressive symptoms among middle-aged adults. Furthermore, the results indicated that individuals with severe edentulism in particular should be assessed for depressive symptoms.

Most long-term studies have demonstrated that oral health problems and a lack of natural teeth may play a role in the development or worsening of depressive symptoms [30, 31]. In a study conducted in Brazil, individuals who experienced tooth loss over a six-year follow-up were at a greater risk of exhibiting depressive symptoms (adjusted prevalence ratio = 1.86; 95% CI: 1.01–3.53) [32]. In the Chilean population, individuals aged 38–74 years with fewer than 20 natural teeth were shown to have higher odds of incident depression at two- and four-year follow-ups [33]. The existence of positive relationships between tooth loss and the occurrence of depressive symptoms was also substantiated by the results of meta-analyses [34] and cross-sectional studies [35, 36]. Matsuyama et al. [36] reported that the effect of tooth loss on depression seemed to be greater in young adults. This phenomenon can be explained by the fact that individuals usually associate the loss of teeth with old age, and consequently, if the problem occurs at a young age, it may exacerbate depressive symptoms. Elderly individuals may treat tooth loss as a natural consequence of old age and adapt their daily life to it more easily than young individuals. The oral health of Poles has remained unsatisfactory for many years compared to that of other European countries, despite a recent trend toward improvement [12, 37, 38]. A total lack of natural teeth, reported by 22.37% of the participants in this study aged 40–64 years, was similar in prevalence to the global prevalence among individuals aged ≥ 45 years, which amounts to 22% [39]. As a result, all negative health outcomes of tooth loss can be expected to appear much earlier in the Polish population.

The mechanism of the relationship between the loss of natural teeth and depressive symptoms has not been investigated or explained in depth thus far. In their long-term study, Yamamoto et al. [30] observed that problems with smiling, laughing, and exposing teeth without embarrassment may cause individuals to isolate themselves and eat alone, which in turn exacerbates their depressive symptoms. Kusama et al. [18] also reported that the deterioration of oral function and orofacial appearance are the main factors contributing to the development of depression due to the loss of natural teeth. Sun et al. [40], based on research conducted among the Chinese population, concluded that dietary dissatisfaction was a contributing factor to the development of depressive symptoms following the loss of teeth. This occurs because food plays an important role in meetings with family and friends and is a medium for maintaining social contact, expressing friendships, and caring for family members. In middle-aged individuals, embarrassment and dissatisfaction with one’s own appearance due to loss of teeth also led to frustration and problems with satisfying activity on the job market, even when dentures were used [17], because removable, unstable dentures did not eliminate certain problems related to aesthetics and oral health. Researchers suggest that depressive symptoms may also be further exacerbated by inflammation of the nervous system resulting from a history of periodontitis or autonomic nerve imbalance caused by oral pain and discomfort [34]. Moreover, Wingfield et al. [41] noted that depression may be related to the state of the oral cavity microbiome. The authors described explicit changes in the composition and amount of specific bacterial taxa in the salivary microbiome in young adults with depression compared to a reference group of individuals without depression.

### Limitations

The limitations of this study include, first and foremost, its cross-sectional design. Moreover, the persons who agreed to participate were volunteers. Thus, they may not be representative of the entire target population. This may result in a sampling bias and limit the generalisability of the results. It should be noted that the relationship between tooth loss and the occurrence of depressive symptoms may be bidirectional [34]. Analysis of depressive symptoms suggested that self-neglect, lowered mood, and lack of energy may cause individuals to neglect oral hygiene and proper eating habits, leading to health problems in the form of oral diseases and tooth loss [35]. Aldosari et al. [31] demonstrated that oral cavity disorders, including tooth loss, were more prevalent among individuals with severe internalization problems. Another limitation that should be mentioned is the potential effect of other factors that have not been included in this study on loss of teeth and the occurrence of depressive symptoms, such as oral hygiene habits [10, 12], number of visits to a dentist [10, 12], well-being [42] or overuse of alcohol [43]. Furthermore, the research tool did not allow for a clinical diagnosis of depression. However, the questionnaire used in this paper has been successfully applied for the assessment of depressive symptoms in other international studies [20]. Conversely, the strengths of this study were the large sample size, uniformity in terms of age, and large number of confounders included in the analysis.

As has been mentioned in the introduction, the prevalence of missing teeth and factors that determine it depend on economic development, access to dental care, social factors and lifestyle, which is why they may differ between countries and regions [8–15]. The obtained results indicated a need for improvement in dental care for middle-aged patients. However, the results may also be used in other European countries with similar living conditions, in particular, in East-Central European countries.

## Conclusions

The results indicated that loss of natural teeth was positively related to the occurrence of depressive symptoms in middle-aged adults. Edentulism showed the strongest relationship with the likelihood of both moderate and severe depressive symptoms. The results of this study have indicated an urgent need to intensify stomatological prophylaxis, education and treatment for middle-aged individuals. Preventing the loss of teeth may potentially reduce the risk of depressive symptoms in this group. This will require the involvement of not only psychologists but also the entire medical community, including dentists, as well as the introduction of appropriate changes in the health care system in Poland aimed at increasing the real availability of dental services.

Better integration of mental and oral health prevention and treatment are recommended. Further longitudinal studies are required to establish the causal and temporal relationship between depressive symptoms and oral health status.

## Data Availability

The datasets used and/or analysed during the current study are available from the corresponding author upon reasonable request.

## Abbreviations

PONS: POlish-Norwegian Study
PURE: Prospective Urban and Rural Epidemiological Study
X: Arithmetic mean
SD: Standard deviation
BMI: Body mass index
OR: Odds ratio
CI: Confidence interval
